# Chemogenetic activation of Gq signaling modulates dendritic development of cortical neurons in a time- and layer-specific manner

**DOI:** 10.3389/fncel.2025.1524470

**Published:** 2025-03-19

**Authors:** Ina Köhler, Lisa Marie Rennau, Adriana Rehm, Julia Große, Steffen Gonda, Andrea Räk, Christian Riedel, Petra Wahle

**Affiliations:** Developmental Neurobiology, Faculty of Biology and Biotechnology, Ruhr University Bochum, Bochum, Germany

**Keywords:** S831 phosphorylation of GluA1, neurite growth, Y1472 phosphorylation of GluN2B, optogenetic stimulation, calcium imaging

## Abstract

Designer receptors exclusively activated by designer drugs (DREADDs) are established tools for modulating neuronal activity. Calcium-mobilizing DREADD hM3Dq has been widely used to enhance neuronal activity. hM3Dq activates the Gq protein signaling cascade and mimics the action of native Gq protein-coupled receptors such as muscarinic m1 and m3 receptors leading to calcium release from intracellular storages. Depolarization evoked by increased intracellular calcium levels is an important factor for neuronal maturation. Here, we used repetitive activation of biolistically overexpressed hM3Dq to increase the activity of individual neurons differentiating in organotypic slice cultures of rat visual cortex. HM3Dq was activated by 3 μM clozapine-N-oxide (CNO) dissolved in H_2_O. Transfectants expressing hM3Dq mock-stimulated with H_2_O served as batch-internal controls. Pyramidal cells and multipolar interneurons were analyzed after treatment from DIV 5–10, DIV 10–20, and DIV 15–20 to investigate if Gq signaling is involved in dendritic maturation. Results show that hM3Dq activation accelerated the maturation of apical dendrites of L2/3 pyramidal cells in the early, but no longer in the later time windows. In contrast, dendritic dimensions of L5/6 pyramidal cells and interneurons were not altered at DIV 10. These findings suggest a growth-promoting role of activated Gq signaling selectively for early postnatal L2/3 pyramidal cells. Unexpectedly, hM3Dq activation from DIV 10–20 reduced the dendritic complexity of L5/6 pyramidal cells and multipolar interneurons. Together, results suggest a role of Gq signaling for neuronal differentiation and support evidence that it may also limit dendritic growth.

## Introduction

Early network activity shapes neuronal morphology and connectivity. Glutamate receptors for instance exert growth-promoting effects on pyramidal cells and multipolar interneurons (Wong and Ghosh, 2002). AMPA receptor isoforms with prolonged opening time and associated type I transmembrane AMPA receptor regulatory proteins promote the growth of pyramidal apical dendrites (Hamad et al., 2011; Hamad et al., 2014). The kainate receptor GluK2 also increases apical complexity in cortical layers 2 and 3 (L2/3) (Jack et al., 2018). In contrast, differentiation of pyramidal cell basal dendrites is regulated by GluN2B-containing NMDA receptors (Wedzony et al., 2005; Gonda et al., 2020). Further, cortical neurons richly express metabotropic receptors for transmitters, neuromodulators, and growth factors. A wealth of studies has characterized metabotropic receptor signaling modulating the electrophysiological features of cortical neurons reporting for instance specific actions of G protein-coupled receptors (GPCRs) in pyramidal cells of the layers 2–6 (Radnikow and Feldmeyer, 2018). Still, less is known about the morphogenetic potential of GPCR signaling elicited by receptors residing in the somatodendritic membrane or aggregating in cilia (Hasenpusch-Theil and Theil, 2021) which can be activated by diffusible factors or by adhesion molecules. Both, growth-promoting and growth-restricting actions have been reported (Guadiana et al., 2013; Sigoillot et al., 2016; Duman et al., 2019; Hamada et al., 2020). In Purkinje cells, a restriction of dendritic growth has been reported with enhanced mGluR1 and mGluR5 signaling possibly via non-canonical pathways (Sirzen-Zelenskaya et al., 2006; Ballester-Rosado et al., 2010). Further, optogenetic stimulation of channelrhodopsin-2 expressing slice cultures results in a reduction of apical dendritic growth of infragranular pyramidal cells (Gonda et al., 2023a). We recently reported that inhibiting pyramidal cells via the Gi/o-coupled DREADD hM4Di delays apical dendritic growth and axonal branching (Gasterstädt et al., 2022) early postnatally, but no longer during a later postnatal time window.

Here, we focus on calcium-mobilizing Gq-mediated signaling using the DREADD hM3Dq. Perinatally in rodent cortex, depolarizing Gq signaling triggers a form of early cortical network activity, for instance, acetylcholine activating muscarinic receptors on subplate cells drive glutamatergic network oscillations in the developing layers (Ghosh et al., 1990; Kilb and Luhmann, 2003; Yang et al., 2016; Dupont et al., 2006; Molnár et al., 2020). Expression of m1/m3 Gq protein is strong in the prenatal rat cortex (Ihnatovych et al., 2002), and cortical pyramidal cells and interneurons continue to express muscarinic receptors postnatally (Levey et al., 1994; Radnikow and Feldmeyer, 2018).

M1-4 muscarinic acetylcholine receptors (mAchR) signaling converges on the ERK1/2 MAP kinase pathway (Rosenblum et al., 2000), and in HEK cells, hMD3q-mediated signaling leads to increased expression of immediate early genes and activates the MAP kinase pathway (Kaufmann et al., 2013). The MAP kinase is a key player in morphological and functional differentiation and neuronal survival (Nourbakhsh and Yadav, 2021). Prenatal activation of hM3Dq in migrating L2/3 pyramidal cells causes premature neuritic branching and laminar mispositioning (Hurni et al., 2017). Depolarizing hM3Dq-mCherry expressing pyramidal cells from postnatal day (P) 5–8 *in vivo* leads to an enhanced glutamatergic output to synaptically connected parvalbumin- and somatostatin-positive interneurons which results in an increased survival at P21 (Wong et al., 2018). Chemogenetic excitation of parvalbumin interneurons or surrounding excitatory neurons does not affect perineuronal net accumulation whereas chemogenetic inhibition of parvalbumin cells reduces net formation (Devienne et al., 2021). Together, similar to optogenetic stimulation, the activation of Gq-mediated signaling seems to have a role in neuronal maturation either by direct action on hM3Dq-expressing neurons or indirectly via the network. Therefore, we investigated if repetitive activation of hMD3q could shape the dendritic trees of pyramidal cells and interneurons of the rat visual cortex.

## Materials and methods

### Preparation of organotypic cultures

Slice cultures were prepared from pigmented Long-Evans rat cortex P1 as described (Gasterstädt et al., 2022). The visual cortex was extracted and cut sagittally into 350 μm thick slices (McIlwain tissue chopper, Ted Pella, Redding, CA, United States). Slices from every individual animal (4–5 animals per batch) were allocated to all experimental conditions run with the batch. Slices were mounted in a coagulate of plasma (2:1 chicken/bovine) and thrombin on coverslips. Cultures were kept at 37°C in roller-tubes with 700 μL semi-artificial medium containing 10% adult horse serum, 25% Hank’s balanced Salt Solution, 50% Eagle’s Basal Medium (Pan-Biotech), 0.5% NeuroCult™ SM1 Neuronal Supplement (STEMCELL Technologies, Cat. #05711), 1 mM L-Glutamine (GIBCO), and 0.65% D-Glucose (Merck). At DIV 2 a mixture of uridine, cytosine-ß-D-arabinofuranoside, and 5-fluoro-2′-deoxyuridin (all from Sigma-Aldrich) suppressed glial differentiation for 24 h.

### Biolistic transfection

Plasmids (Table 1) were prepared as endotoxin-free solutions (EndoFree Plasmid Maxi Kit, Qiagen, Cat. #12362) and stored as 1 μg/μl stocks at −20°C. Gene gun cartridges were produced by coating 7 mg gold microparticles (1 μm diameter; MaTeck GmbH) with 10–15 μg plasmid DNA. For calcium imaging, pGP-CMV-GCaMP6m was transfected with pAAV-hSyn-hM3D(Gq)-mCherry to identify the transfected neurons and classify the neuron type. Since large fluorophore tags may influence receptor trafficking to proper cellular localizations, pcDNA5/FRT-HA-hM3D(Gq) was transfected for immunohistochemical localization of hM3Dq receptors. For morphometry, complete cytosolic labeling of dendrites and axons was achieved by co-expressing pcDNA5/FRT-HA-hM3D(Gq) with pEGFP-N1. For Western Blot analysis, pcDNA5/FRT-HA-hM3D(Gq) transfected cultures were used.

**Table 1:** Plasmids.

Plasmid	Promoter	Source, application	Catalog number
pEGFP-N1	CMV	Clontech, Heidelberg, Germany; used to completely label the neurons	cat# 632370
pGP-CMV-GCaMP6m	CMV	(Chen et al., 2013) (gift from Douglas Kim), for calcium imaging	RRID: Addgene_40754
pAAV-hSyn-hM3D(Gq)-mCherry	Human synapsin 1	Gift from Bryan Roth, for calcium imaging	RRID: Addgene_50474
pcDNA5/FRT-HA-hM3D(Gq)	CMV	(Armbruster et al., 2007) (gift from Bryan Roth), for morphometry, Western Blot	RRID: Addgene_45547
pcDNA3.1/hChR2(H134R)-EYFP	CMV	(Zhang et al., 2007) (gift from Karl Deisseroth), for Western Blot	RRID: Addgene_20940

Biolistic transfection for chemogenetic experiments (Helios Gene Gun, Bio-Rad Laboratories) was performed at DIV 4 or DIV 14 with 180 psi helium pressure. High co-expression rates of biolistic transfections have been previously confirmed (Wirth et al., 2003; Hamad et al., 2011).

For the optogenetic experiments, biolistic transfection of channelrhodopsin-2-EYFP was conducted at DIV 8 using the pcDNA3.1/hChR2(H134R)-EYFP construct (Gonda et al., 2023a; Gonda et al., 2023b).

### Chemogenetic stimulation

DREADDs are GPCRs harboring two-point mutations (Y3.33C and A5.46G) which decrease the affinity for their natural agonist acetylcholine and the muscarinic agonist carbachol and instead yield a high affinity for the synthetic ligand CNO (Armbruster and Roth, 2005; Armbruster et al., 2007; Bonaventura et al., 2019). HM3Dq has been most frequently used with acute stimulation for behavioral studies in juvenile and adult animals. We reported that DREADDs are also useful for developmental studies. As has been done in our previous study (Gasterstädt et al., 2022), cultures expressing hMD3q in single neurons were stimulated from DIV 5–10, and from DIV 10–20, and DIV 15–20 once daily with CNO at 3 μM final concentration in the medium (CNO stock solution dissolved in ddH_2_O; ENZO Life Sciences). Control cultures also expressed hMD3q but were mock-stimulated with ddH_2_O. This way, CNO was the only experimental variable. The concentration of 3 μM CNO has been reported to yield near-maximal activation of hMD3q in HEK cells (Armbruster et al., 2007). The medium was changed every second day to minimize drug accumulation. In a previous study, EGFP-transfected neurons were stimulated with CNO, and no effect was observed on dendritic differentiation (Gasterstädt et al., 2022) suggesting an absence of CNO-mediated side effects.

For cFos experiments, cultures were supplemented for 1.5 h with 5 μM CNO which is still specific for the DREADDs and does not activate native mAchR (Gomez et al., 2017). The drug has been used at a higher concentration and enhances diffusion into the tissue slice in order to activate neurons and elicit cFos transcription. CFos is transiently expressed yielding a nuclear staining. We knew from earlier studies (Gonda et al., 2023a) that cFos is well detectable after 1.5 h of optogenetic stimulation. We now show that cFos was well detectable in hMD3q transfectants.

### Optogenetic stimulation

Optogenetic stimulation at 0.5 Hz frequency is effective in altering the growth of pyramidal cell apical dendrites and interneurons (Gonda et al., 2023a). Cultures were kept in a dark room to reduce exposure to white light. Stimulation was performed using a custom-designed illumination setup with 24 individually switchable 465 nm LEDs (Osram Oslon SSL80; Lumitronix), controlled via an Arduino unit as detailed (Gonda et al., 2023a). The distance between the cultures and the LEDs was 12 mm. Light intensity was measured with a photodiode (S130VC; Thorlabs) and a power meter (PM100D; Thorlabs) at the level of the cultures in the culture tubes resulting in an intensity of 0.7 mW/mm^2^. From DIV 11–15, cultures underwent three daily rounds of stimulation at a frequency of 0.5 Hz with 70 ms and 140 ms pulse duration (Gonda et al., 2023a).

### Immunostaining

Detailed information about antibodies is listed in Table 2. For morphometry, cultures were fixed with 4% paraformaldehyde in 0.1 M phosphate buffer pH 7.4 for 30 min at 37°C, rinsed, and permeabilized with 0.3% Triton X-100 in phosphate buffer for 30 min. Cultures were blocked with 5% BSA in TBS and incubated in mouse anti-EGFP or mouse anti-mCherry antibodies for 12–24 h. Then, biotinylated goat anti-mouse was incubated for 2 h, followed by ABC reagent (Vector Laboratories Inc.) for 2 h, and HRP reaction with 3,3-*diaminobenzidine* (DAB; Sigma-Aldrich). The staining of the DAB product was intensified for 30 s with 1% OsO_4_ (Sigma-Aldrich). For mounting, cultures were dehydrated and coverslipped with DEPEX (Sigma-Aldrich). Subcellular distribution of hM3Dq-HA was demonstrated with mouse anti-HA.11 overnight followed by donkey anti-mouse Alexa Fluor 568. For the detection of cFos protein, cultures were incubated with rabbit anti-cFos and mouse anti-HA.11, followed by donkey anti-rabbit Alexa Fluor 488 and donkey anti-mouse Alexa Fluor 568, and nuclear counterstain with DAPI. Proportions of hM3Dq-transfected cells with cFos-positive nuclei per culture were determined.

**Table 2:** Antibodies.

	Species	Source	Cat. no., RRID	Method, dilution	Antigen (kDa)
Primary antibody					
cFos	Rabbit	Santa Cruz Biotechnology	Cat# sc-52, RRID: AB_2106783	IHC, 1:500	–
EGFP	Mouse	Sigma-Aldrich	Cat# G6795, RRID: AB_563117	IHC, 1:1000	–
HA.11	Mouse	BioLegend	Cat# 901515, RRID: AB_2565334	IHC, 1:750	–
β-actin	Mouse	Sigma-Aldrich	Cat# A1978, RRID: AB_476692	WB, 1:1000	40
GAD 65/67	Mouse	Enzo Life Sciences	Cat# ADI-MSA-225-E, RRID: AB_2039129SA-225E	WB, 1:1000	65/67
GluA1	Rabbit	Alomone Labs	Cat# AGC-004, RRID: AB_2039878	WB, 1:1000	100
GluA1 phS831	Rabbit	Rockland	Cat# 612-401-C82, RRID: AB_11181727	WB, 1:1000	100
GluN2B	Rabbit	Millipore	Cat# 06-600, RRID: AB_310193_310193	WB, 1:1000	180
GluN2B phY1472	Rabbit	Sigma-Aldrich	Cat# M2442, RRID: AB_262150	WB, 1:1000	180
Synapsin-1	Mouse	Synaptic Systems	Cat# 106001a, RRID: AB_2617071	WB, 1:1500	78
Synaptotagmin-2	Rabbit	Synaptic Systems	Cat# 105123, RRID: AB_2199465	WB, 1:1000	70
Synaptophysin 1 (p38)	Mouse	Synaptic Systems	Cat# 101011C2, RRID: AB_10890165	WB, 1:1000	38
PSD 95	Rabbit	Synaptic Systems	Cat# 124002, RRID: AB_887760	WB, 1:1000	95
Secondary antibodies					
Anti-mouse biotinylated	Goat	Agilent	Cat# E0433, RRID: AB_2687905	IHC, 1:750	–
Anti-mouse Alexa 568	Donkey	Abcam	Cat# ab175472, RRID: AB_2636996	IHC, 1:1000	–
Anti-rabbit Alexa 568	Donkey	Abcam	Cat# ab175470, RRID: AB_2783823	IHC, 1:1000	–
Anti-rabbit Alexa 488	Donkey	Abcam	Cat# ab150073, RRID:AB_2636877	IHC, 1:1000	–
Anti-mouse-AP	Rabbit	Dako A/S	Cat# D0314	WB, 1:5000	–
Anti-rabbit-AP	Goat	Agilent	Cat# D0487, RRID: AB_2617144	WB, 1:2000	–
ABC reagent	–	Vector Laboratories Inc.	Cat# PK-7100, RRID: AB_2336827	As recommended	–

### Image acquisition

Fluorescent images were captured using a Leica TSC SP5 confocal microscope with both 10× and 40× objectives (1.1 NA, 1,024 × 1,024 pixels). Global adjustments for contrast, brightness, color intensity, saturation settings, and scale bars were made using ImageJ (MacBiophotonics). Surface reconstruction of labeled neurons was performed using Imaris software version 10.1.1 (Oxford Instruments).

### Calcium imaging

Neurons transfected with hM3Dq-mCherry and GCaMP6m were recorded at DIV 10–11 and between DIV 15–20 to confirm the calcium-mobilizing function reported for hM3Dq (Armbruster et al., 2007) and to validate our plasmid construct. Cultures were adjusted to oxygenated HEPES-ACSF for 1 h in the roller incubator. Then, calcium events were recorded in regions of interest placed over the somata of transfectants with a Leica TCS SP5 confocal microscope using a × 10 objective and sampling at 1,400 Hz and 2.7 frames/s as reported earlier (Hamad et al., 2014; Jack et al., 2018). Roller cultures regularly display calcium events driven by endogenous spontaneous activity. This basal activity level was recorded for every cell for 5 min, and baseline fluorescence (F_o_) was calculated for every cell by averaging the 20 frames with the lowest intensity. Then, CNO in HEPES-ACSF was superfused at a final concentration of 3 μM, and cells were recorded for 10 min. After a 2 min wash-out cells were recorded for another 5 min. The maximal calcium event amplitude was detected during the pre-, wash-in, and after the wash-out recording periods. The duration of events (defined as rise and decline to baseline fluorescence) was measured at half-maximal width. Raw data were delivered in the form of a linear 16-bit intensity scale versus time and analyzed using MacBiophotonics ImageJ software. Normalizing ΔF/F_o_ was done to allow comparison of multiple recordings done with cultures from 3 independent preparations and was used to plot the data with the control set to 1.

### Protein blots

Culture lysates and blots were performed as described (Engelhardt et al., 2018) with OTCs from at least 3 individual preparations that were transfected with hM3Dq and stimulated with CNO. For chronic stimulation, cultures were daily treated from DIV 5–10 and 10–20 with 3 μM CNO. For acute stimulation at DIV 10, cultures were stimulated once with 5 μM CNO and harvested after 1 h. For control, hM3Dq transfected cultures were mock-stimulated with ddH_2_O. In addition, we tested in the acute and chronic stimulation at DIV 10 if CNO application can alter protein expression in the absence of hM3Dq. Initially, CNO has been considered inert. However, CNO becomes metabolized to clozapine and related metabolites which can interfere with neuronal signaling (Löffler et al., 2012; MacLaren et al., 2016). We have recently shown that prolonged stimulation with CNO does not affect the morphogenesis of EGFP-expressing neurons (Gasterstädt et al., 2022). Thus, as a complementary approach, we submitted EGFP-only transfected cultures to CNO treatment followed by protein blot analysis. Optogenetically stimulated cultures were harvested 6 h after the last stimulation block at DIV 15 to remain consistent with the previous studies (Gonda et al., 2023a; Gonda et al., 2023b).

Lysates were prepared with standard RIPA buffer (300 mM NaCl, 100 mM Tris–HCl pH 7.4, 10 mM EDTA, 10 mM NaF, 200 μM Na_3_VO_4_), 10% phosphatase inhibitor cocktail (PhosSTOP, Roche), 5% protease inhibitor cocktail (cOmplete, Roche), 4% SDS, 1% Nonidet P40 (AppliChem), 25 mM deoxycholic acid (Roth) and 2 mM serine protease inhibitor phenylmethylsulfonyl fluoride (Sigma). The samples were homogenized and denatured at 80°C for 10 min. Lysates (1 OTC/lane) were separated on 10% SDS-PAGE and transferred to nitrocellulose (Whatman, 0.45 μm) using a tank blotter (TE Series Transphor electrophoresis unit, Hoefer). Membrane strips were placed over the molecular weight range of the target proteins of interest this way detecting several proteins of distinct kDa in every lysate. Membranes were blocked in 5% BSA in TBS for 1.5 h at room temperature. Antibodies against the following proteins were incubated overnight: GluN2B, GluN2B phTyr1472, GluA1, GluA1 phS831, synapsin-1, synaptophysin 1 (p38), PSD 95, synaptotagmin-2 (Syt 2) and GAD-65/67. Visualization of protein bands was done with alkaline phosphatase-conjugated secondaries against mouse and rabbit and reaction with 5-bromo-4-chloro-3-indolyl-phosphate/nitroblue tetrazolium (Promega). Protein bands were scanned, and band intensities were assessed with ImageJ followed by normalization to the intensity of the β-actin band stained in each lane. Gel by gel, the normalized band intensities of the proteins of the control lysates were set to 1.

### Morphometry

A quantitative assessment was performed for pyramidal cell dendrites and dendrites of multipolar interneurons at DIV 10 and DIV 20. Neurons DAB-immunostained for the co-expressed EGFP were 3D reconstructed at DIV 10 and DIV 20 using a Zeiss Axioskop50 at 1000× magnification linked with a camera (Microfire, Optronics) to the Neurolucida system (*MicroBrightField,* Inc.) by trained observers blinded to conditions. All reconstructions were crosschecked for correctness and classification by a second observer, also blinded to conditions. Pyramidal cells and multipolar interneurons were classified by established criteria of dendritic and axonal patterns (Hamad et al., 2011; Jack et al., 2018; Gasterstädt et al., 2020; Gonda et al., 2023b). Laminar assignment of pyramidal cells was performed as follows. Apical dendrites of pyramidal neurons of L2/3 reach into L1 whereas those of L5/6 end in middle layers. Thick and thin tufted large L5 pyramidal cells were too rarely transfected and were not reconstructed. For the data sets at DIV 20, multipolar interneurons were classified as basket cells and non-basket cells. The non-basket cells comprise translaminar-projecting bitufted and arcade axon cells forming collaterals with regular-sized delicate boutons. Basket cells form locally dense or horizontally projecting axons studded with irregular-sized boutons (Gonda et al., 2023b). Reconstructed neurons were analyzed using the NeuroExplorer software (MicroBrightField, Inc.). For basal dendrites of pyramidal cells and dendrites of interneurons, mean values for length and segments were computed and assessed with a Sholl analysis. The maximum branch order of the apical dendrites was determined by using a centrifugal approach that started at the soma. The order increases by 1 at each branchpoint. The Strahler order was applied as a centripetal ordering scheme (Horton, 1945; Strahler, 1957). The Strahler number, defined as the maximum Strahler order of the apical dendrite, was determined and the percentage of Strahler numbers per condition was computed.

### Statistical analysis

Bar and box plots, Sholl plots, and statistical analyses were done with Sigma Plot 12.3 (Systat). Non-parametric ANOVA on ranks tests was performed for calcium imaging data with appropriate corrections for multiple testing (Bonferroni’s or Dunn’s test). Mann–Whitney rank sum tests were used for testing the CNO stimulation versus H_2_O mock stimulation.

## Results

### Cortical neurons have stable hM3Dq protein expression

**Figure 1:**
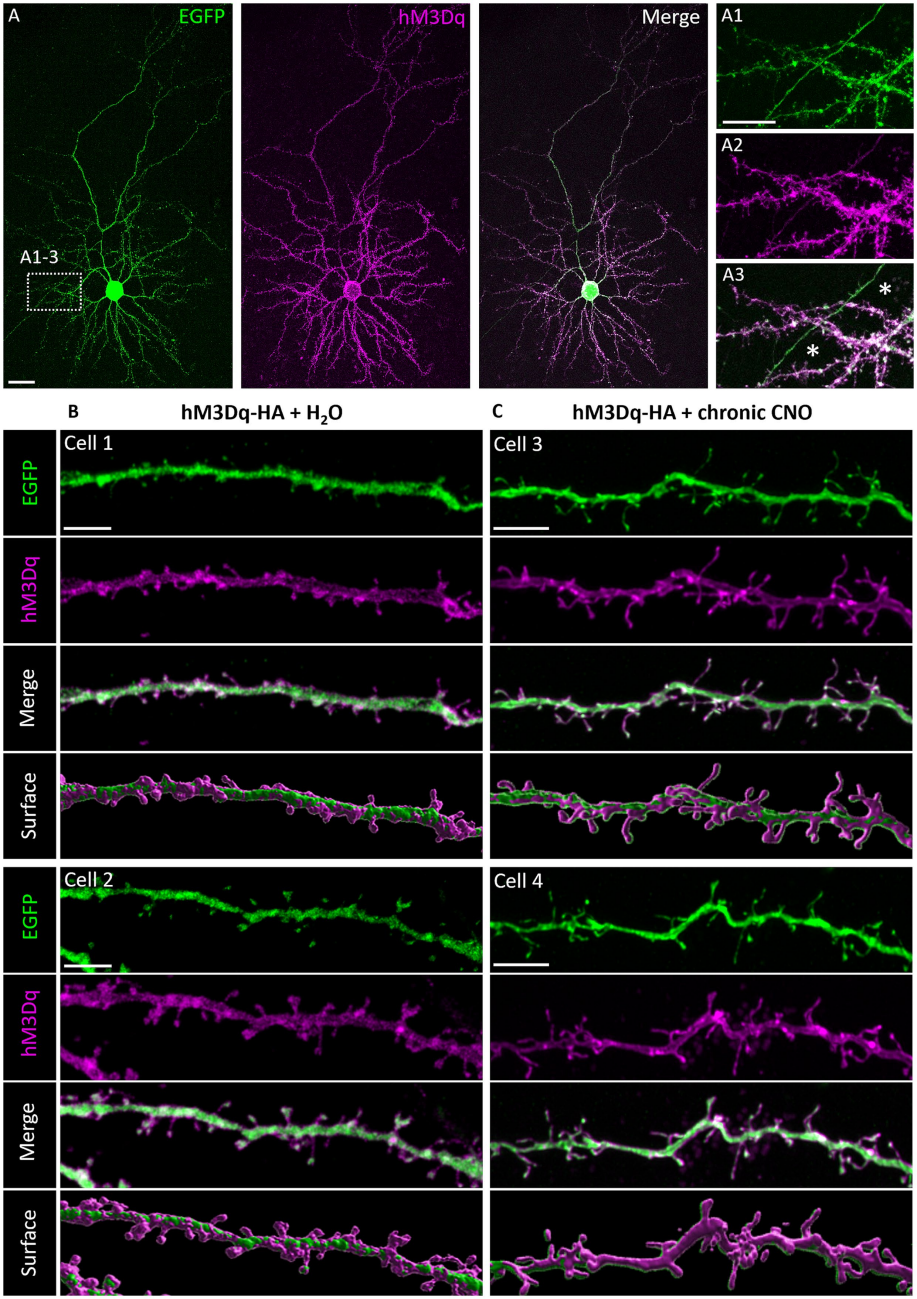
Immunofluorescent staining of pyramidal neurons expressing HA-tagged hM3Dq. **(A)** Co-transfection of plasmids encoding EGFP and HA-tagged hM3Dq visualized with mouse anti-HA/Alexa-568 and merged. **(A1–A3)** Higher magnification of dendrites with a collateral of the axon of this cell (asterisks). Note that the collateral harbored less hM3Dq-HA than the dendrites. **(B)** HM3Dq receptor was stably expressed in pyramidal cells stimulated once daily from DIV 10–20 with H_2_O or **(C)** with CNO; apical oblique and basal dendritic segments of different cells were shown with the single channels and merged, and surface reconstruction (Imaris). Note that hM3Dq-HA immunoreactivity was in dendritic shafts, spine necks, and heads. Scale: 20 μm for **(A)**; 10 μm for **(A1–A3)**; and 5 μm for **(B,C)**.

**Figure 2:**
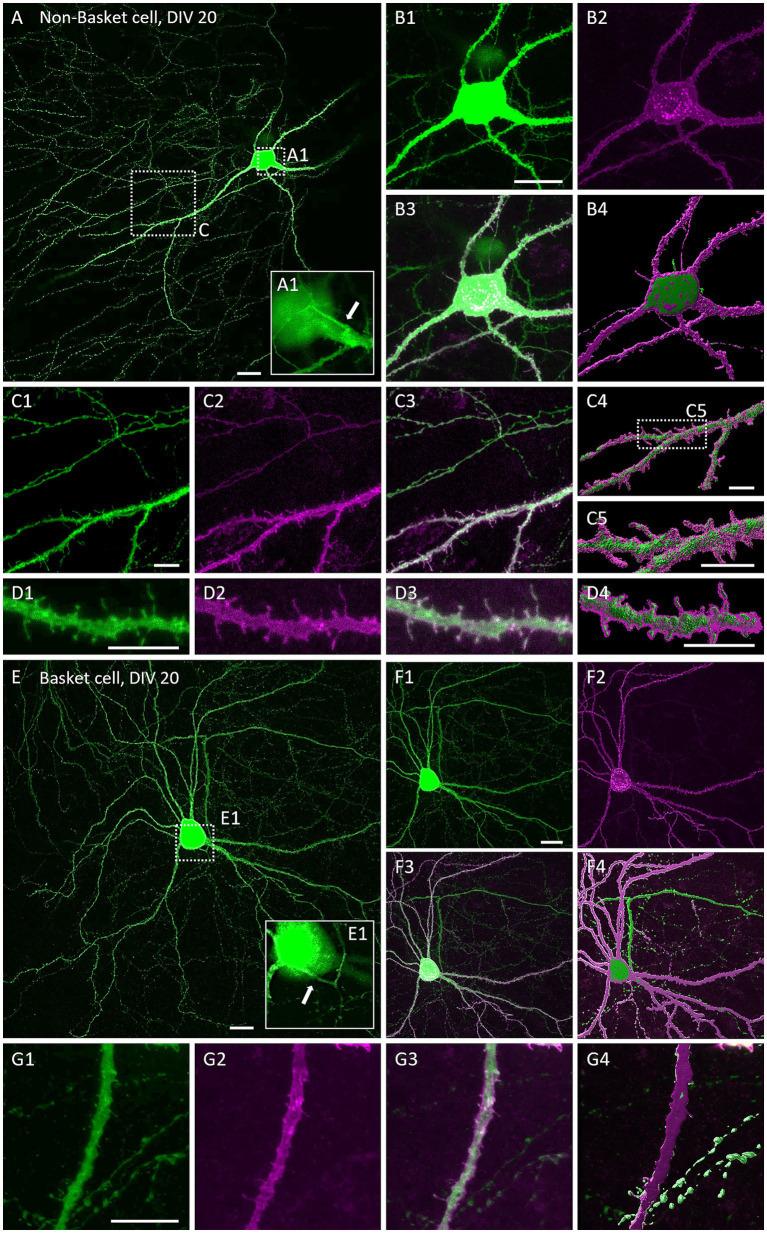
Immunofluorescent staining of multipolar cortical interneurons expressing hM3Dq-HA. Co-transfection of plasmids encoding EGFP and HA-tagged hM3Dq visualized with mouse anti-HA/Alexa-568 and merged. **(A–D)** Non-basket neuron. **(A)** Overview of a non-basket cell at DIV 20. **(A1)** Magnification of the soma, white arrow indicates the axon origin. **(B1–B3)** Close-up of the non-basket cell soma shown in **(A)**. **(B4)** Surface reconstruction of EGFP and hM3Dq-HA immunoreactivity with the Imaris software. **(C1–C3)** Close-up of a dendrite and axon collateral of the same neurons, and **(C4,C5)** the surface reconstruction. **(D1–D3)** Magnification of a dendritic segment and **(D4)** the surface reconstruction. **(E–G)** Basket neuron. **(E)** Overview of a basket cell at DIV 20. **(E1)** Magnification of the soma, white arrow indicates axon origin. **(F1–F3)** EGFP and hM3Dq expression in soma, dendrites, and axon of the basket cell. **(F4)** Surface reconstruction. **(G1–G4)** Magnification of a dendritic segment with varicose axon collaterals. Scale: 20 μm for **(A,B,E,F)** and 10 μm for **(C,D,G)**.

The natural Gq-linked muscarinic receptors m1, m3_,_ and m5 are expressed in the visual cortex and are mainly located postsynaptically (Levey et al., 1991; Amar et al., 2010; Elhusseiny and Hamel, 2000), but also presynaptically in pyramidal cells (de Vin et al., 2015) and interneurons (Lawrence et al., 2015). To confirm that overexpression of hM3Dq and localization in dendrites can be achieved and is stable, we transfected neurons at DIV 4 with an HA-tagged hM3Dq to enable proper receptor trafficking. Neurons were co-transfected with EGFP to completely visualize the neuron. At DIV 20, hMD3q-HA immunopositive pyramidal neurons displayed strong labeling in dendrites. A certain amount of immunoreactive material remained somatic suggestive of retention in the ER or Golgi apparatus (Figure 1A). HM3Dq occurred weakly in axons and axon collaterals (Figures 1A1–A3). Higher magnification revealed hM3Dq-HA immunoreactivity in dendritic shafts and spine necks and heads (Figures 1B,C) and staining appeared membrane-associated as expected for a receptor. Labeling intensities appeared similar in hMD3q-HA-transfected neurons that were mock-stimulated (Figure 1B) or stimulated with CNO (Figure 1C) from DIV 10–20. In summary, hM3Dq-HA staining suggested that cortical neurons can express and traffic hM3Dq protein to the plasma membrane. Also, repetitive CNO stimulation did not alter the expression and localization of hM3Dq-HA. Interneurons transfected with hM3Dq-HA displayed comparable localization of the receptor. Non-basket (Figures 2A–D) and basket cells (Figures 2E–G) have strong labeling in dendrites and spines. Also here, some ER retention is obvious (Figures 2B,F). Interneuronal axons and axon collaterals expressed much less immunoreactivity (Figures 2C,G).

### HM3Dq activation depolarizes cortical neurons

**Figure 3:**
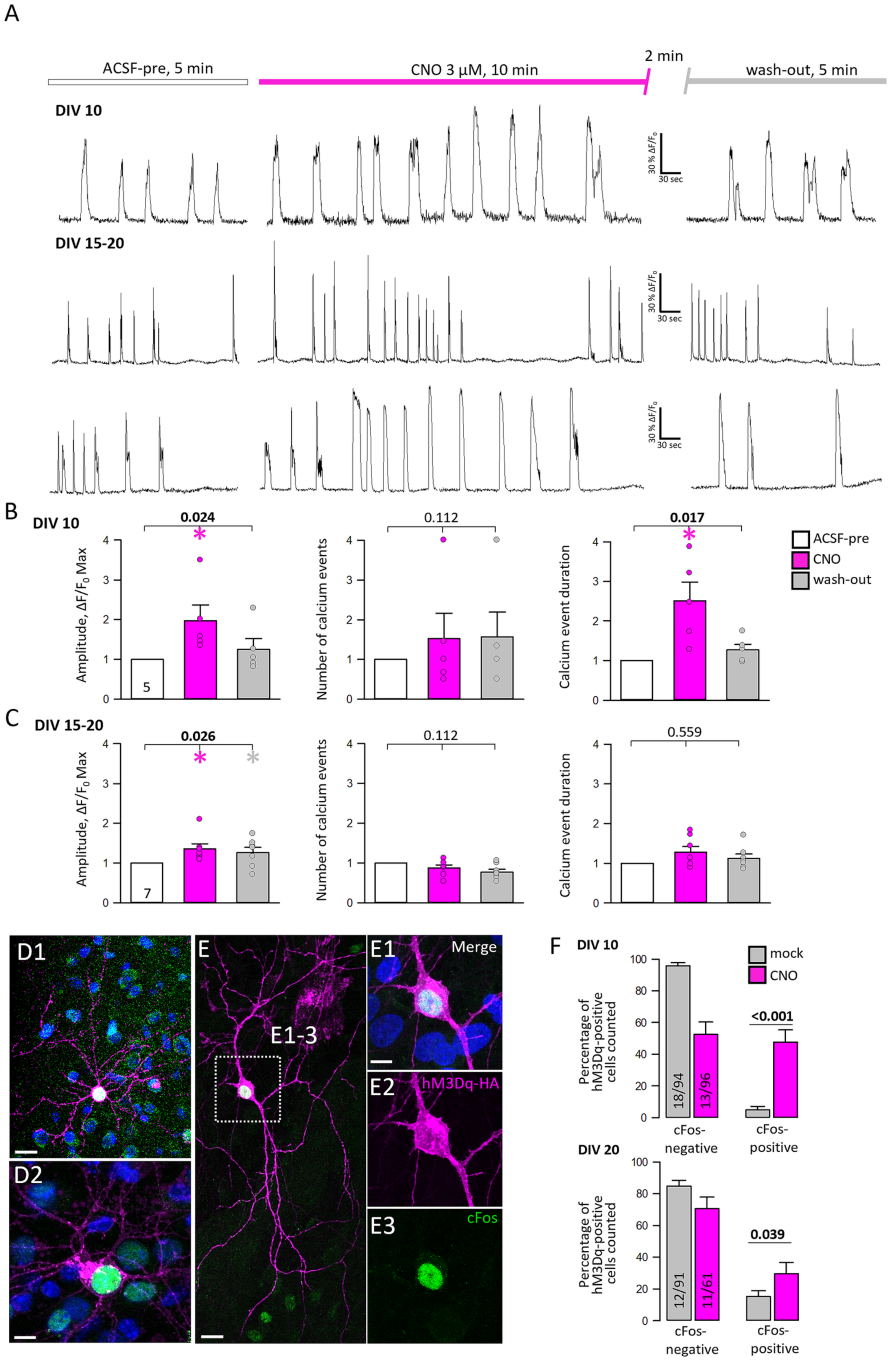
The activation of hM3Dq enhances neuronal activity and immediate early gene expression. **(A)** Representative traces of spontaneous calcium events of three selected neurons were recorded at DIV 10 (upper trace) and DIV 15–20 (lower two traces). **(B)** Quantitative analysis (mean ± s.e.m.) at DIV 10–11, and **(C)** at DIV 15–20 of calcium event amplitude and frequency in ACSF (white), under CNO (pink), and after wash-out (dark gray). The number of neurons analyzed is given in the light gray bar. Statistics with ANOVA on ranks, ACSF-pre versus CNO, and wash-out. Significant *p*-values in bold, colored asterisks indicate where significant differences have been detected. **(D,E)** HM3Dq-HA transfected neurons were stained against cFos 1.5 h after acute stimulation with 5 μM CNO. **(D1,D2)** cFos-positive pyramidal cells. **(E)** cFos positive interneuron shown with single channels in **(E1–E3)**. The merge also includes DAPI staining **(E1)**. Scale bar: 20 μm for **(D1,E)**, 10 μm for **(D2,E1–E3)**. **(F)** Quantitative analysis (mean ± s.e.m) showing the proportions of cFos-negative and -positive transfected neurons per OTC after acute mock or CNO stimulation at DIV 10 and DIV 20. The number of analyzed OTCs from 4 independent preparations and the number of assessed neurons is given in the bar. Statistics: Mann–Whitney rank sum test.

To confirm a functional expression of hMD3q calcium imaging was performed at DIV 10–11 and DIV 15–20 of mCherry-tagged hM3Dq and GCaMP6m (Figure 3). Cultures are spontaneously active. Therefore, the baseline activity of transfectants was monitored for 5 min, followed by a wash-in of 3 μM CNO and recording for 10 min. After a 2 min wash-out period, the cells were recorded for another 5 min in ACSF only. At DIV 10–11, calcium event amplitudes were significantly increased after activation of hM3Dq (Figure 3B, left graph), and declined after wash-out. The number of events was not increased (Figure 3B, middle), but the calcium event duration was significantly prolonged (Figure 3B, right; Supplementary Table S1). At DIV 15–20, the amplitude was subtly increased and remained slightly increased after the CNO wash-out (Figure 3C, left graph). The number and duration of calcium events were no longer increased by CNO. To check if hM3Dq activation can elicit activity-dependent gene expression, cFos staining was performed (Figures 3D,E). HM3Dq-positive pyramidal cells (Figures 3D1,D2) and transfected interneurons show cFos-immunopositive nuclei after CNO stimulation (Figures 3E1–E3). Transfected neurons were counted at DIV 10 and DIV 20. At DIV 10, the activation of hM3Dq increased the percentage of cFos-positive transfectants (Figure 3F, upper graph). However, at DIV 20, the activation of hM3Dq led only to a small increase of cFos-positive cells over the control level. In summary, hM3Dq activation increased calcium event amplitudes and elicited activity-dependent gene expression in immature neurons.

### Chemogenetic and optogenetic stimulation reduce GluA1 levels

MAchR signaling enhances the excitability of pyramidal cells and evokes dendritic calcium spikes and action potentials (Fernández de Sevilla et al., 2021) suggesting that hM3Dq stimulation will enhance the synaptic output of transfected neurons into the network. Our biolistic method aimed at a sparse transfection of neurons to allow error-free reconstruction. Earlier studies report that GluK2 transfectants activated by endogenously released glutamate are highly active and act as drivers for network activity (Jack et al., 2018). Further, a given “driver” cell in L2/3 will reliably activate only a small number of “followers” within a 200 μm radius, and the small number of reliably driven neurons has been shown to evoke only a moderately elevated population response (Meyer et al., 2018). Moreover, the synchronous activity of channelrhodopsin-2 transfected and non-transfected neurons was minimal during light stimulation suggesting that the transfectants do not substantially increase network activity (Goold and Nicoll, 2010). Together, this suggested that we can elicit a moderate level of network activity which then may elicit changes in protein expression.

We selected proteins known to increase postnatally and to be regulated by bioelectric activity, and related to the development of interneurons (GAD-65/67, synaptotagmin-2), synapses (synaptophysin 1, synapsin-1, PSD 95) and associated with pyramidal cell dendritic differentiation (GluN2B, Y1472-phosphorylated GluN2B, GluA1, and S831-phosphorylated GluA1) (Patz et al., 2003; Hamad et al., 2011; Engelhardt et al., 2018; Jack et al., 2018; Gonda et al., 2023a). For instance, GluA1 was of interest because these calcium-permeable subunits are relevant for activity-dependent synaptic plasticity and particularly relevant for interneuronal dendrite growth (Hamad et al., 2011). Further, we looked at the S831 phosphorylation of GluA1 which is mediated by CamKII and PKC. Phosphorylation at this residue indicates enhanced trafficking and synaptic plasma membrane localization, renders conformational stability of the C-terminus, enhances single-channel conductance and open probability, and supports long-term potentiation (LTP) (Sanz-Clemente et al., 2013; Charter and Goda, 2014). Further, GluN2B receptors were of interest because they are modulated by several Gq-containing GPCRs (Yang et al., 2014) and changes in synaptic strength mediated by mAchR and NMDA receptor signaling both converge on AMPA receptor trafficking (Sumi and Harada, 2023).

**Figure 4:**
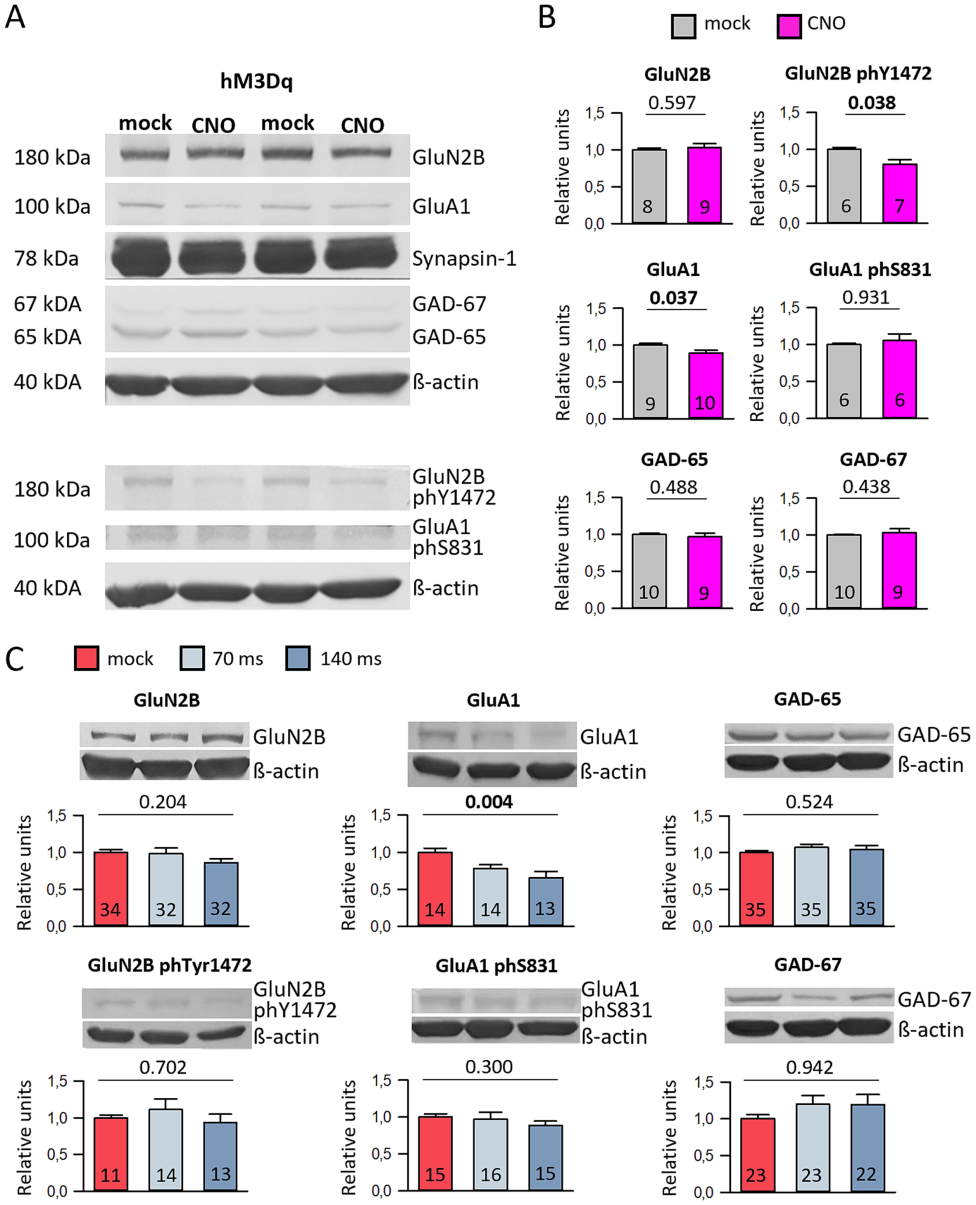
Chemogenetic and optogenetic stimulation reduces GluA1 protein levels. **(A)** Representative Western Blot images are presented. HM3Dq-transfected cultures daily stimulated with mock or CNO from DIV 10–20 were harvested at DIV 20. The culture lysates were divided, and samples from the same lysate were applied in two lanes to blot for GluN2B and GluA1, as well as phosphorylated Y1472 GluN2B and phosphorylated S831 GluA1. All protein bands shown are from the same gel **(B)**. The plots represent data from hM3Dq-transfected cultures at DIV 20, showing relative protein levels normalized to β-actin. The average protein level in the control groups is set to 1. The number of cultures analyzed is indicated in the bars. Statistical analysis was performed using the Mann–Whitney rank sum test, with *p*-values provided above the plots. **(C)** Representative Western blot bands and bar graphs for optogenetically stimulated cultures (stimulated at 0.5 Hz for 70 ms and 140 ms) at DIV 15. The relative protein levels, normalized to β-actin, are displayed, with the average of the control groups set to 1. The number of cultures analyzed is indicated in the bars. Statistical analysis was conducted using ANOVA on ranks, with p-values shown above the plots. Please note that panels **(B,C)** include a selection of the blotted proteins. For more details about all the blotted proteins, please refer to Supplementary Tables S2, S3.

As controls, mock-stimulated hM3Dq-transfected cultures were used as well as CNO-stimulated cultures transfected with EGFP only, as reported previously (Gasterstädt et al., 2022). In Supplementary Table S2, all protein values were reported normalized to the mock-stimulated hM3Dq condition. Acute stimulation for 1 h at DIV 10 did not alter the levels of the selected proteins but reduced the S831 phosphorylation of GluA1 (Supplementary Table S2A). Chronic stimulation from DIV 5–10 did not alter the levels of the proteins analyzed (Supplementary Table S2B). After chronic stimulation from DIV 10–20, the total GluA1 protein was reduced as was the Y1472 phosphorylation of GluN2B (Figures 4A,B; Supplementary Table S2C). The reduction of GluA1 protein was also observed in the DIV 10–15 channelrhodopsin-2 cultures optogenetically stimulated at 0.5 Hz with 70 ms and 140 ms duration (Figure 4C; Supplementary Table S3). Together, this suggested that the activation of hMD3q could well depolarize the transfected neurons comparable to an optogenetic stimulation. The activation with both tools was sufficiently strong to evoke cFos expression, but not strong enough to evoke changes in the amounts of selected proteins except for about 15–20% reduction of total GluA1 protein.

### Activation of hM3Dq shapes pyramidal differentiation

**Figure 5:**
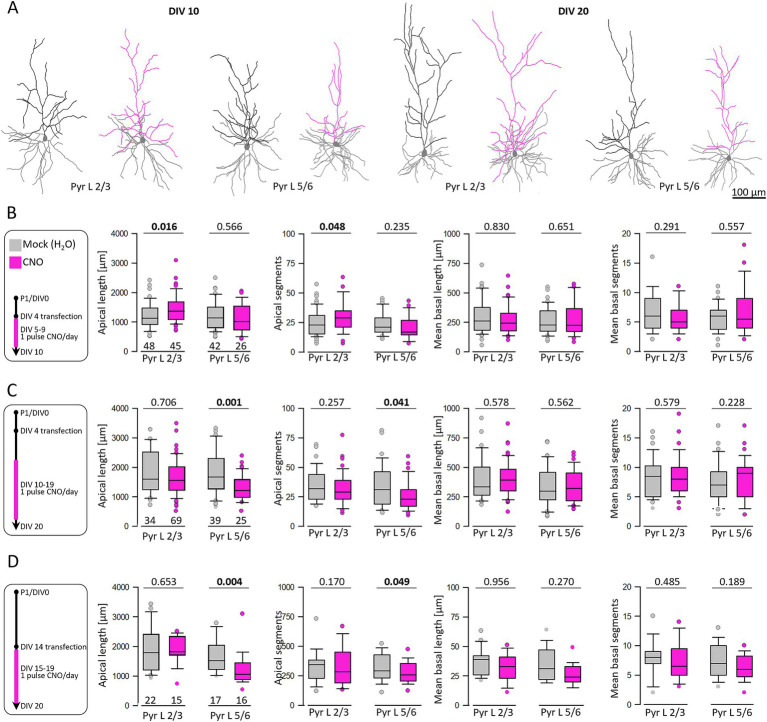
Activation of hM3Dq can increase and reduce pyramidal apical dendritic complexity in age- and layer-dependent fashion. **(A)** Skeletal drawings of reconstructed pyramidal cells at DIV 10 and DIV 20. Basal dendrites are in gray; apical dendrites are in black for control and in pink for stimulated neurons. **(B)** Stimulation protocol and morphometric analysis of L2/3 and L5/6 pyramidal cells at DIV 10. **(C)** Stimulation protocol and morphometric analysis of L2/3 and L5/6 pyramidal cells at DIV 20. **(D)** Analysis of L2/3 and L5/6 pyramidal cells at DIV 20 after transfection at DIV 14 and CNO stimulation from DIV 15–20. The number of analyzed neurons is given below the box plots. Statistics: Mann–Whitney rank sum test H_2_O as control versus CNO, *p*-values are reported with the significant difference in bold lettering.

Previous data have shown that increased calcium even amplitude evoked by overexpression of AMPA and kainate receptor subunits increased apical dendritic complexity of pyramidal neurons (Hamad et al., 2011; Hamad et al., 2014; Jack et al., 2018). Accordingly, our working hypothesis has been that increased calcium amplitudes through hM3Dq activation drive dendritic growth. Therefore, neurons were co-transfected with hM3Dq and EGFP at DIV 4 and DIV 14, respectively, treated daily with CNO or H_2_O from DIV 5–10, DIV 10–20, and DIV 15–20. After EGFP staining, pyramidal cells were reconstructed (Figure 5). Skeletal drawings of reconstructed pyramidal cells treated from DIV 5–10 and DIV 10–20 are shown (Figure 5A). At DIV 10, hM3Dq activation significantly increased apical dendritic length and segment numbers of L2/3 pyramidal cells (Figure 5B). Strikingly, the action was different in the later time window. Stimulation from DIV 10–20 (Figure 5C) reduced apical dendritic length and branching of L5/6 pyramidal cells. Growth of basal dendrites of pyramidal cells in either laminar compartment was not affected by hM3Dq activation at either time point.

The growth-limiting effect in infragranular neurons might be due to the longer exposure to CNO. To narrow down on the critical period for this effect we analyzed neurons transfected at DIV 14 followed by CNO stimulation from DIV 15–20 (Figure 5D). The late postnatal stimulation for 5 days yielded significantly shorter and less branched L5/6 apical dendrites. The average values (mean ± s.e.m.) at the three time windows were reported in Supplementary Table S4.

**Figure 6:**
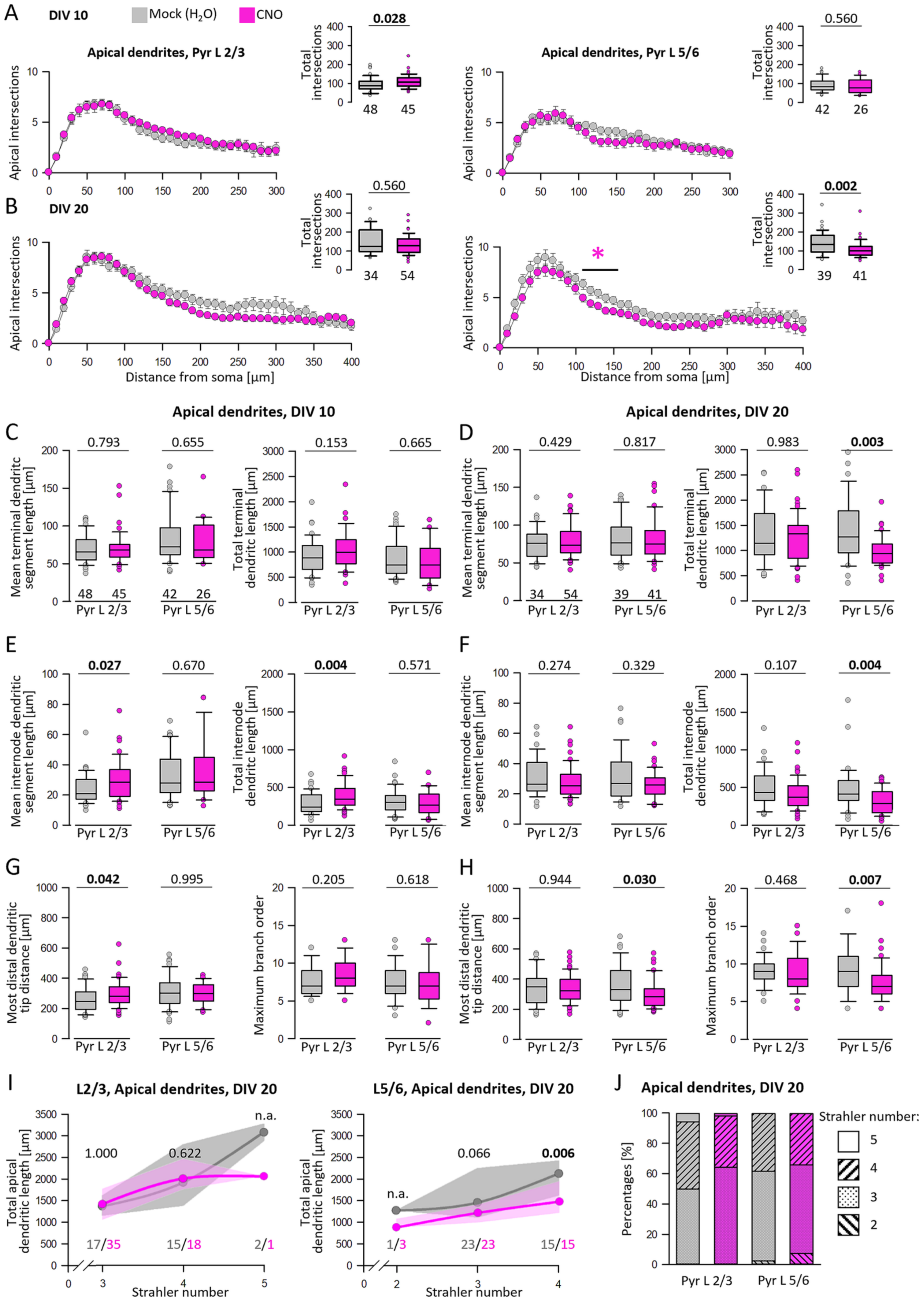
Analysis of apical dendrites regarding terminal and internode structure. **(A)** Sholl analysis and total intersections of L2/3 and L5/6 pyramidal cell apical dendrites at DIV 10. **(B)** Sholl analysis and total intersections of L2/3 and L5/6 pyramidal cell apical dendrites at DIV 20. Colored asterisks indicate where significant differences have been detected. **(C,E,G)** Analysis of DIV 10 apical dendrites regarding terminal and internode mean and total segment length, the most distal apical tip from the soma, and maximum branch order starting from the soma. **(D,F,H)** Analysis of DIV 20 apical dendrites regarding terminal and internode mean and total segment length, the most distal apical tip from the soma, and centrifugal maximum branch order. **(I)** L2/3 and L5/6 pyramidal cell apical lengths were grouped according to their apical Strahler number. **(J)** Percentages of apical Strahler numbers for L2/3 and L5/6 pyramidal cells. The number of analyzed neurons is given below the box plots, and in **(I)**, the numbers are given below curves. Statistics: Mann–Whitney rank sum test H_2_O as control versus CNO, *p*-values are reported. n.a., not assessed because there are too few neurons with this Strahler number.

Sholl analyses were performed to analyze dendritic complexity in more detail (Figure 6). At DIV 10, the number of total intersections of CNO-treated L2/3 apical dendrites was increased (Figure 6A, left graph, inset) as if growth increased over the entire tree and not within particular bins. A detailed analysis of DIV 10 apical dendrites showed that neither the average length of terminal segments nor the total terminal length was affected (Figure 6C). Rather, the increased length and complexity of L2/3 pyramidal cells resulted from longer internode segments (Figure 6E). This is supported by the on average larger distance between the soma and the distalmost branch tip of the apical tuft (Figure 6G). The complexity of L5/6 pyramidal cell apical dendrites was not altered by CNO at DIV 10 (Figure 6A, right graph, Figures 6C,E,G).

At DIV 20, reduced apical dendritic complexity of L5/6 pyramidal cells was detectable in the Sholl analysis within 120–190 μm distance from the soma (Figure 6B, right graph). Also, the number of total intersections was reduced (Figure 6B, right graph, inset). In contrast, DIV 20 L2/3 pyramidal cells remained at the control level. At DIV 20, the apical dendrites of CNO-treated L5/6 pyramidal cells exhibited a reduction in both total terminal and internode lengths. However, the average segment length was not significantly reduced. This argued for a subtle decrease in the number of segments and, consequently, total length (Figures 6D,F). As a result, the reduced distance between the most distal branch tip and the soma as well as the reduction of maximal branch order supported the idea of shorter apical dendrites of L5/6 pyramidal cells (Figure 6H).

A more detailed analysis of branch order using the centripetal Strahler ordering analysis revealed that DIV 20 pyramidal cells primarily fall within Strahler numbers 3 and 4 (Figures 6I,J). Notably, in the group of neurons with Strahler number 4, the total apical dendritic length of CNO-stimulated L5/6 pyramidal cells was significantly reduced compared to the control group. Furthermore, control pyramidal cells from L 2/3 and L5/6 exhibited slightly higher percentages of higher Strahler orders than CNO-stimulated neurons (Figure 6J). In summary, hM3Dq-mediated activity can either increase or reduce the apical dendritic complexity of L2/3 and L5/6 pyramidal cells depending on the stimulation period.

### Activating hM3Dq signaling subtly impairs interneuronal dendritic development

**Figure 7:**
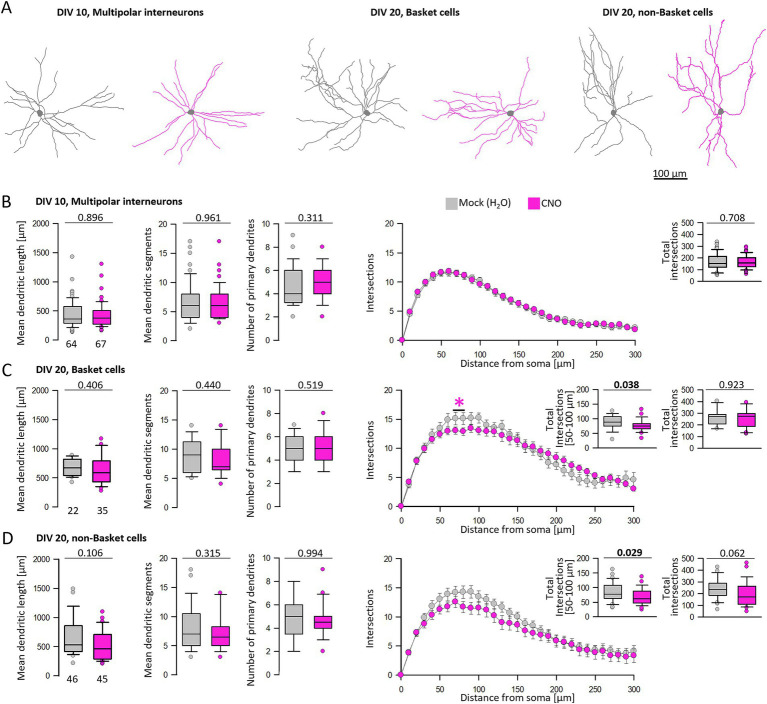
Analysis of multipolar interneurons after hM3Dq stimulation at DIV 10 and 20. **(A)** Skeletal drawings of interneurons at DIV 10 and DIV 20. Mock stimulated interneurons in gray, CNO treated interneurons in pink. **(B)** Dendritic parameters and Sholl analysis of DIV 10 multipolar interneurons. **(C)** Analysis of basket cell dendrites at DIV 20. **(D)** Analysis of non-basket cell dendrites at DIV 20. In Figures 7C,D, small insets compare the total intersections up to 300 μm distance and the intersections within 50–100 μm distance to the soma. The number of analyzed neurons per condition is given below the box plots. Colored asterisks indicate where significant differences have been detected. Statistics: Mann–Whitney rank sum test mock versus CNO, *p*-values are reported.

At DIV 10, hM3Dq-mediated calcium signaling did not affect the dendritic complexity of interneurons, and the Sholl curves were almost identical (Figures 7A,B). In cultures older than DIV 10, interneuron types become recognizable allowing the classification of cells as basket cells with local axons and horizontal collaterals, and non-basket cells with vertically translaminar axonal projections such as bitufted neurons and neurons with arcade axons using established criteria (Figure 7A) (Gonda et al., 2023b). The average dendritic length and segment numbers as well as the number of primary dendrites of basket cells were not altered after CNO treatment compared to the control condition (Figure 7C). However, the Sholl analysis revealed a mild impairment on the dendritic complexity within a 50–100 μm distance from the soma where the curve of CNO-stimulated basket cells undershoots the curve of the mock-stimulated basket cells. In the bins of 50–100 μm distance to the soma, the number of intersections was significantly reduced (Figure 7C, inset).

Of the non-basket cells, dendritic length, segment numbers, and number of primary dendrites of CNO-stimulated cells were not different from control measures (Figure 7D). Again, the Sholl analysis revealed a statistical trend (*p* = 0.062) toward a reduction of total branching (Figure 7D, inset), and the curve of CNO-stimulated non-basket cells was consistently undershooting the control curve. In the bins of 50–100 μm distance to the soma, the number of intersections was significantly reduced (Figure 7D, inset). In summary, the dendritic complexity of basket and non-basket interneurons was reduced by hM3Dq-induced Gq signaling during the DIV 10–20 major phase of maturation of cortical interneurons.

## Discussion

HM3Dq has been proven to be a reliable tool in neurophysiological and behavioral studies in adolescent and adult mammals. Here, we show that hM3Dq is useful also for developmental approaches. Trafficking of GPCRs, such as muscarinic receptors, between cytoplasmic compartments and the plasma membrane is tightly regulated (Bernard et al., 2006). Early postnatal cortical neurons stably express and traffic hM3Dq to dendrites with membrane-associated immunopositivity detectable in dendritic shafts and spines. The expression pattern resembles the reported postsynaptic localization of Gq-coupled muscarinic receptors m1 and m3 (Mrzljak et al., 1993; Gu, 2003; Gulledge et al., 2009; Levey et al., 1994). Besides, the protein clustered in the cytosol was suggestive of ER retention. However, even cytosolic muscarinic receptors may function, since both, surface and internal m1 receptors have been shown to support the induction of LTP in hippocampal neurons with the latter acting via the MAP kinase pathway (Anisuzzaman et al., 2013). HM3Dq-HA immunoreactivity was also in axons and presynaptic terminals of pyramidal cells and of interneurons which suggested that hM3Dq becomes transported to the axonal domain. In fact, m3 receptors have a major role in the presynaptic inhibition of excitatory synaptic transmission in rat CA1 pyramidal cells (de Vin et al., 2015), and in developing basket cells activated during theta and gamma oscillations, presynaptic mAchR signaling reduces the calcium influx and prevents vesicle depletion (Lawrence et al., 2015).

Concerning pharmacokinetics, we exposed our cultures only once daily with a rather low-to-moderate dosage of CNO and medium exchange every second day, although we cannot tell to which degree hM3Dq will become internalized and recycled to the membrane. It is known that muscarinic receptors undergo agonist-dependent internalization. For instance, Chinese hamster ovary cells internalize about 40% of m3 receptors upon exposure to the cholinergic agonist carbachol for 4 h, and receptors recycle back to the membrane 1 h after carbachol wash-out (Thangaraju and Sawyer, 2011). In striatal neurons of hypercholinergic rats, m2 receptors are nearly absent from the plasma membrane with a significant accumulation in the cytoplasm indicating that an excess of the ligand blocks m2 trafficking to the membrane (Liste et al., 2002). So, it has been promising to see that even after repetitive stimulation over 10 days hM3Dq immunopositivity remained apparently membrane-associated in the transfectants, and that layer- and age-specific effects on dendritic complexity were seen despite the rather low dose of CNO.

Moreover, calcium imaging of hM3Dq-expressing neurons showed an increase in the calcium event amplitude in neurons aged DIV 10–11 and DIV 15–20. Further, at DIV 10–11 the duration of the calcium events was prolonged. The frequency of events was not modulated by hM3Dq activation. An enhanced calcium event amplitude has been previously reported to efficiently promote pyramidal cell apical dendritic growth. For instance, an overexpression of the GluA2 and GluA3 AMPA receptor subunits and, similarly, of type 1 transmembrane AMPA receptor regulatory protein γ-8 in immature cortical neurons leads to higher calcium event amplitudes and a subsequent increase of apical dendritic complexity (Hamad et al., 2011; Hamad et al., 2014). Further, the hM3Dq-induced calcium mobilization was sufficient to elicit cFos expression in the activated neurons (Sheng and Greenberg, 1990) at DIV 10, and less so at DIV 20, possibly because the maturation of inhibition has attenuated the efficiency of the CNO stimulation in the later postnatal time window.

Activation of hMD3q transfectants did not alter the levels of most of the selected proteins. For instance, one possible result could have been an upregulated expression of GAD-65/67 which is known to increase with enhanced network excitation and/or activity-mediated enhancement of neurotrophin signaling (Hendry and Jones, 1988; Welker et al., 1989; Patz et al., 2003). The result argued against the induction of pathological activity upon stimulation of a few hM3Dq transfectants. Further, the CNO-induced downregulation selectively of the S831 phosphorylation but not of total GluA1 at DIV 10 argues against the possibility that hM3Dq stimulation has evoked pathological activity. The latter has been shown to reduce always both, total GluA1 and phosphorylated S831 GluA1 levels (Russo et al., 2013; Lopes et al., 2013). Albeit significant, the effect on S831 phosphorylation appears moderate presumably because the fraction of phosphorylated GluA1 subunits varies from 12–50%, and only a subset of synapses harbors GluA1 (Diering et al., 2016). Nevertheless, the reduced S831 phosphorylation suggested that GluA1-containing AMPA receptors are removed from the synaptic membrane which would increase the threshold for LTP induction and would rather promote the induction of LTD often reported for mAchR stimulation.

The CNO-induced reduction of total GluA1 protein at DIV 20 was intriguing. In earlier work, biolistic overexpression of GluA1 subunits from DIV 5–10 has been shown to increase the dendritic complexity of multipolar interneurons, but the subunits had no effect on pyramidal cell apical dendrites (Hamad et al., 2011). It was suggestive to assume that this reduction led to the observed growth reduction at DIV 20 of both, basket cells and non-basket cells as revealed by the Sholl analyses. The assumption is supported by our parallel approach with a DIV 10–15 optogenetic 0.5 Hz stimulation. It revealed an even stronger downregulation of GluA1 with 70 ms and 140 ms light pulse duration, and this stimulation also reduces the dendritic complexity of interneurons (Gonda et al., 2023b). Also, mAchR-stimulation has been shown to induce expression of the plasticity-regulated immediate early protein ARC (Teber et al., 2004) which contributes to the removal of calcium-permeable AMPA receptors from potentiated synapses (Rao and Finkbeiner, 2007). The S831 phosphorylation was neither altered with CNO treatment nor with optogenetic stimulation. Total protein, surface level, and phosphorylation level are not necessarily correlated (Mao et al., 2015) and it is well possible that the preservation of S831-phosphorylated GluA1 occurs in pyramidal cells whereas the reduction of total GluA1 might have happened in the interneurons which represent only 20% of the cortical neurons. Reducing the amount of GluA1 may be viewed as circuit-stabilizing maturation because it may reduce the aggregation of GluA1 homomers and attenuate high conductance states, both of which have been associated with unstable synaptic states (Benke and Traynelis, 2019).

Stimulation of muscarinic receptors has been reported to cause long-term depression (LTD). However, it depends on context, region, and input, since LTD of synaptic transmission in L2/3 of the visual cortex is expressed in the monocular segment and requires activation of m3 receptors whereas postsynaptic potentials of L2/3 pyramidal cells of the binocular region undergo LTP via activation of m1 receptors and PLC (McCoy et al., 2008). The mAchR-induced LTD of NMDAR-mediated synaptic transmission involves the release of calcium from intracellular stores and finally the internalization of NMDARs via endocytosis (Jo et al., 2010). The DIV 10–20 CNO treatment reduced the Y1472 phosphorylation of GluN2B. Phosphorylation at this site enhances the trafficking and anchoring of GluN2B in synaptosomal membranes (Goebel et al., 2005). Recordings from CA3 pyramidal cells in organotypic slices have shown that m1 receptor signaling leads to an activation or a tyrosine phosphatase which depresses NMDA responses (Grishin et al., 2005). A lower phosphorylation at this residue enhances the interaction of GluN2B receptors with the clathrin adaptor AP-2 which enables endocytosis of GluN2B-containing receptors and lateral movement out of the synapse to extrasynaptic sites (Tovar and Westbrook, 1999; Li et al., 2002; Chen and Roche, 2007). Our result suggested that stimulating Gq signaling between DIV 10–20 leads to a reduction of synaptic GluN2B-containing receptors which might enhance the coincident exchange to GluN2A this way stabilizing circuits and restricting synaptic plasticity.

With regard to the morphometry, the growth-promoting effects are presumably evoked by the direct action of hMD3q signaling in the transfected neurons which is supported by the age-, and cell-type-specific effects. At DIV 10, apical dendritic growth was enhanced only in L2/3 pyramidal neurons. Neurons of infragranular layers and multipolar interneurons were not responding. A comparable result has been obtained with overexpression of AMPA and kainate receptor subunits indicating that both approaches can evoke a level of depolarization sufficient to increase apical dendritic complexity, and further, that apical dendrites of supragranular neurons are most plastic during this early time window (Hamad et al., 2011; Jack et al., 2018).

The reason why supragranular pyramidal cells and in particular their apical dendrites are more plastic and responsive to stimulation is not fully clear. Being ontogenetically younger and in an earlier period of maturation than infragranular cells may be one reason. Further, apical and basal dendrites grow with different mechanisms (Gonda et al., 2020). It may further be related to the behaviorally highly relevant calcium potentials displayed by apical dendrites (Larkum et al., 2022). Besides, cortical layers differ in local wiring, activity patterns, and receptor equipment including for instance, specific mAchR-induced firing patterns (Mendoza-Halliday et al., 2024; Hagger-Vaughan et al., 2024).

In contrast, hMD3q signaling from DIV 10–20 reduced apical dendritic growth of L5/6 pyramidal neurons. Comparable growth-limiting effects have been observed in L5/6 pyramidal cells after 0.5 Hz optogenetic stimulation from DIV 10–15 resulting in a significant reduction of both, the length of dendritic internodes and the maximum branch order (Gonda et al., 2023a). We now observed similar effects after DIV 10–20 hM3Dq activation. Analysis of the Strahler order showed that the symmetry of the dendritic trees remained mostly unchanged by the stimulation. Our values are comparable to Strahler orders of apical dendrites reported earlier (Vormberg et al., 2017). Furthermore, at DIV 20, the dendritic complexity of CNO-stimulated basket and non-basket interneurons was reduced as was the dendritic complexity of optogenetically stimulated interneurons at DIV 15 (Gonda et al., 2023b), both coincident with a reduction of GluA1 total protein.

However, we also found differences between optogenetic and chemogenetic stimulation. The activation of ChR2 already reduces apical dendritic complexity of L5/6 pyramidal cells at DIV 10 (Gonda et al., 2023a) while hM3Dq stimulation until DIV 10 had no such effect. In our hands, the chemogenetic approach with calcium release from internal storage seemed less forceful than depolarization via the opening of a microbial sodium channel at the plasma membrane. Calcium influx via NMDA receptors results in a translational protein expression profile which differed from the translational response evoked by internal release (Ramakrishna et al., 2024). In addition, at DIV 10, the maturing inhibitory system might be able to keep the mild hMD3q-evoked depolarization of individual neurons in check but is not yet efficient enough to counter ChR2-evoked neuronal firing.

Reorganization of apical dendrites resulting in shortening and loss of branches has already been described. In the rat visual cortex, callosally projecting pyramidal cells of L5 reduce apical dendritic complexity around the 10th postnatal day (Koester and O’Leary, 1992). Similarly, L4 spiny stellates retract their apical dendrite and adopt a non-polar shape in the visual cortex of early postnatal cats (Vercelli et al., 1992) and monkeys (Lund et al., 1991). In ferrets, visual deprivation decreases the proportion of remodeled L4 spiny stellates indicating that apical dendritic pruning is an activity-dependent process (Callaway and Borrell, 2011). The change in apical dendritic complexity has physiological consequences. The shorter the dendrite, the less excitable the cell is and the more resistant is it to burst firing (Galloni et al., 2020).

Basal dendritic growth was not altered by hMD3q signaling. This was unexpected because many Gq-coupled GPCRs can modulate NMDA receptor activity via PKC and src kinase signaling (Yang et al., 2014), and GluN2B signaling drives basal dendritic growth until DIV 10 (Gonda et al., 2020). However, of the muscarinic receptors, m1 rather than m3 receptors have been reported to enhance GluN2-mediated currents (Yang et al., 2014).

## Conclusion

Results show that Gq signaling via hM3Dq has a morphogenetic role in developing cortical neurons. The two subsets of pyramidal cells analyzed responded in an age-dependent manner. Early on, Gq activation promoted apical dendritic development of immature L2/3 pyramidal neurons. Later, in more mature pyramidal neurons of L5/6 and multipolar interneurons, Gq activation reduced apical dendritic complexity. Results suggested that Gq signaling might counter the strong growth-promoting action for instance of BDNF during the critical period and stabilize the postsynaptic compartment of the developing cortical circuit.

## Data Availability

The original contributions presented in the study are included in the article/Supplementary material, further inquiries can be directed to the corresponding author.
