# Supplementary material for: Chemogenetic activation of Gq signaling modulates dendritic development of cortical neurons in a time- and layer-specific manner
Source: Front Cell Neurosci. 2025 Mar 19;19:1524470. doi: 10.3389/fncel.2025.1524470 (PMC11962018; doi:10.3389/fncel.2025.1524470)
Supplement: Supplementary file 1 [file Data_Sheet_1.PDF]

Supplementary Material

Supplementary Table 1. Raw values of calcium imaging experiments with hM3Dq and GCaMP6m transfected pyramidal neurons at DIV 10-11 and DIV15-20. Calcium events were recorded in regions of interest placed over the somata of transfected cells using a Leica TCS SP5 confocal microscope with a ×10 objective. Sampling was conducted at a rate of 1400 Hz and 2.7 frames per second. Baseline activity levels were recorded for each cell over 5 minutes, and baseline fluorescence (Fo) was calculated by averaging the 20 frames with the lowest intensity. Subsequently, CNO in HEPES-ACSF was superfused at a final concentration of 3 µM, and cells were recorded for an additional 10 minutes. After a 2-minute wash-out period, recordings continued for another 5 minutes. The maximal amplitude of calcium events was detected during the ACSF , CNO wash-in, and wash-out recording periods. The duration of these events, defined as the time taken to rise and decline back to baseline fluorescence, was measured at half-maximal width. Raw data were provided as a linear 16-bit intensity scale versus time and were analyzed using MacBiophotonics ImageJ software. To facilitate comparison across multiple recordings from cultures derived from three independent preparations, the ΔF/Fo was normalized.

Supplementary Table 1. Raw values of calcium imaging experiments with hM3Dq and GCaMP6m transfected pyramidal neurons at DIV 10 and DIV15-20

Age, (N of OTCs)	Amplitude ($\Delta F/F_0$)			Number of calcium events			Calcium event duration (sec)		
	ACSF-pre	CNO	Wash-out	ACSF-pre	CNO	Wash-out	ACSF-pre	CNO	Wash-out
DIV 10 (5)	6.60 ± 1.49	12.87 ± 3.34	7.66 ± 1.66	2.5 ± 0.6	3.1 ± 1.0	3.2 ± 0.86	7.14 ± 1.47	16.2 ± 2.29	9.13 ± 2.22
DIV 15-20 (7)	8.19 ± 1.79	10.89 ± 2.17	10.52 ± 2.37	15.6 ± 3.4	14.0 ± 8.9	12.4 ± 3.3	3.97 ± 0.81	5.55 ± 1.59	4.64 ± 1.09

Supplementary Table 2. Quantitative results of Western blot analysis of hM3Dq or GFP transfected cultures stimulated with CNO or H2O at DIV10 and DIV20 under chronic and acute conditions. Culture lysates and blots were conducted as described by Engelhardt et al. (2018), using cultures from at least three individual preparations that were transfected with hM3Dq and stimulated with CNO. (A) Acute stimulation at DIV 10, the cultures received a single treatment of 5 µM CNO and were harvested after one hour. For chronic stimulation, cultures were treated daily with 3 µM CNO from DIV 5 to 10 (B) and DIV 10 to 20 (C). As a control, hM3Dq-transfected cultures were mock-stimulated with ddH2O. Additionally, a second control was added where EGFP-transfected cultures were stimulated with CNO (see A and B). Protein bands were scanned, and their intensities were analyzed using ImageJ, followed by normalization to the intensity of the β-actin band in each lane. For each gel, the normalized band intensities of the proteins in the control lysates were set to a value of 1.

Supplementary Table 2. Quantitative results of Western blot analysis of hM3Dq or GFP transfected cultures stimulated with CNO or H_2O at DIV10 and DIV20 under chronic and acute conditions.

Age, Condition	GluN2B	GluN2B Y1472	GluA1	GluA1 phS831	Synapsin 1	P38	PSD-95	Syt 2	GAD 67	GAD 65
**(A) DIV 10 acute, 1 h**										
GFP + CNO	1.152 ± 0.123 (10)	1.150 ± 0.076 (13)	1.161 ± 0.109 (14)	1.213 ± 0.157 (5)	1.170 ± 0.091 (14)	0.931 ± 0.059 (12)	1.113 ± 0.082 (10)	1.184 ± 0.092 (14)	1.143 ± 0.057 (15)	1.054 ± 0.058 (15)
hM3Dq + H_2O	1.000 ± 0.049 (21)	1.000 ± 0.031 (23)	1.000 ± 0.047 (24)	**1.000 ± 0.041 (14)**	1.000 ± 0.043 (24)	1.000 ± 0.041 (22)	1.000 ± 0.047 (10)	1.000 ± 0.032 (24)	1.000 ± 0.015 (24)	1.000 ± 0.028 (24)
hM3Dq + CNO	0.971 ± 0.093 (18)	1.077 ± 0.068 (20)	1.082 ± 0.071 (22)	**0.857 ± 0.053 (12)**	0.992 ± 0.075 (22)	1.042 ± 0.123 (19)	1.112 ± 0.080 (9)	1.036 ± 0.078 (22)	1.087 ± 0.058 (22)	1.016 ± 0.070 (22)
Anova on Ranks	0.660	0.251	0.599	**0.035**	0.369	0.731	0.380	0.349	0.170	0.841
**(B) DIV 10 chronic**										
GFP + CNO	1.071 ± 0.057 (13)	1.157 ± 0.064 (10)	1.168 ± 0.109 (13)	n. d.	1.013 ± 0.058 (12)	1.112 ± 0.153 (6)	1.071 ± 0.056 (10)	1.025 ± 0.049 (13)	1.047 ± 0.029 (11)	0.988 ± 0.041 (13)
hM3Dq + H_2O	1.000 ± 0.016 (12)	1.000 ± 0.037 (10)	1.000 ± 0.049 (12)	n. d.	1.000 ± 0.038 (11)	1.000 ± 0.046 (6)	1.000 ± 0.032 (10)	1.000 ± 0.024 (12)	1.000 ± 0.016 (11)	1.000 ± 0.032 (12)
hM3Dq + CNO	0.941 ± 0.071 (14)	1.188 ± 0.138 (9)	0.983 ± 0.096 (15)	n. d.	0.981 ± 0.080 (11)	0.951 ± 0.067 (6)	1.048 ± 0.084 (9)	0.875 ± 0.215 (15)	0.970 ± 0.038 (13)	0.906 ± 0.064 (15)
Anova on Ranks	0.210	0.213	0.466		0.965	0.722	0.492	0.107	0.202	0.583
**(C) DIV 20 chronic**										
hM3Dq + H_2O	1.000 ± 0.030 (8)	**1.000 ± 0.021 (6)**	**1.000 ± 0.023 (9)**	1.000 ± 0.021 (6)	1.000 ± 0.041 (10)	1.000 ± 0.045 (8)	1.000 ± 0.022 (6)	1.000 ± 0.041 (10)	1.000 ± 0.053 (10)	1.000 ± 0.022 (10)
hM3Dq + CNO	1.036 ± 0.052 (9)	**0.840 ± 0.052 (7)**	**0.894 ± 0.039 (10)**	1.054 ± 0.092 (6)	0.935 ± 0.050 (10)	0.957 ± 0.078 (8)	1.019 ± 0.056 (6)	1.018 ± 0.055 (9)	1.032 ± 0.053 (9)	0.975 ± 0.047 (9)
Mann-Whitney test	0.597	**0.038**	**0.037**	0.931	0.820	0.574	0.699	0.838	0.488	0.438

Supplementary Table 3. Quantitative results of Western blot analysis of ChR2 transfected cultures stimulated with 0.5 Hz for 70 ms or 140 ms at DIV15. Culture lysates and blots were conducted as described by Engelhardt et al. (2018), using cultures from at least three individual preparations that were transfected with Channelrhodopsin-2 and stimulated DIV 11-15. Cultures underwent three daily rounds of stimulation at a frequency of 0.5 Hz with 70 ms and 140 ms pulse duration (Gonda et al., 2023a). Protein bands were scanned, and their intensities were analyzed using ImageJ, followed by normalization to the intensity of the β-actin band in each lane. For each gel, the normalized band intensities of the proteins in the control lysates were set to a value of 1.

Supplementary Table 3. Quantitative results of Western blot analysis of ChR2 transfected cultures stimulated with 0.5 Hz for 70 ms or 140 ms at DIV15

Age, Condition	GluN2B	GluN2B phY1472	GluA1	GluA1 phS831	Synapsin 1	P38	PSD-95	Syt 2	GAD 67	GAD 65
**DIV 15**										
mock	1.000 ± 0.039 (34)	1.000 ± 0.036 (11)	**1.000 ± 0.054 (14)**	1.000 ± 0.045 (15)	1.000 ± 0.353 (16)	1.000 ± 0.029 (35)	n. d.	1.000 ± 0.054 (17)	1.000 ± 0.058 (23)	1.000± 0.031 (35)
70 ms	0.983 ± 0.075 (32)	1.114 ± 0.142 (14)	**0.783 ± 0.049 (14)**	0.973 ± 0.095 (16)	1.129 ± 0.129 (16)	0.998 ± 0.197 (34)	n. d.	0.882 ± 0.083 (18)	1.203 ± 0.117 (23)	1.074 ± 0.045 (35)
140 ms	0.859 ± 0.055 (32)	0.939 ± 0.111 (11)	**0.656 ± 0.085 (13)**	0.885 ± 0.062 (15)	0.997 ± 0.119 (16)	0.939 ± 0.204 (33)	n. d.	0.936 ± 0.074 (17)	1.195 ± 0.135 (22)	1.048 ± 0.049 (35)
Anova on Ranks	0.204	0.702	**0.004**	0.300	0.647	0.323		0.392	0.942	0.524

Supplementary Table 4. HM3Dq transfected pyramidal cells stimulated with CNO at DIV5-10, DIV10-20 and DIV15-20. Measures of apical and basal dendrites of hM3Dq transfected pyramidal neurons of L2/3 and L5/6 stimulated with CNO versus batch-internal hM3Dq-expressing neurons stimulated with H_2O as control at DIV 5-10, DIV 10-20 and DIV 15-20. Given is the mean ± s.e.m., and n, the number of cells analyzed. Statistics: Mann-Whitney rank sum test H2O control versus CNO. ADL, apical dendritic length (µm); BDL, mean (per cell) basal dendritic length (µm); segments, number of dendritic segments.

Supplementary Table 4. HM3Dq transfected pyramidal cells stimulated with CNO at DIV5-10, DIV10-20 and DIV15-20

	Pyramidal cells in L2/3		Pyramidal cells in layers L5/6	
Stimulation, Condition<br>(Number of batches)	ADL (n)<br>Segments	BDL<br>Segments	ADL (n)<br>Segments	BDL<br>Segments
**DIV 5-10**				
Control (7)	**1211 ± 63** (48)<br>**24.7 ± 1.4**	295 ± 22<br>6.6 ± 0.5	1200 ± 75 (42)<br>23.9 ± 1.6	260 ± 18<br>5.5 ± 0.4
CNO	**1446 ± 71** (45)<br>**29.9 ± 1.8**	266 ± 18<br>5.9 ± 0.4	1122 ± 95 (26)<br>21.0 ± 1.9	278 ± 29<br>6.7 ± 0.8
Mann-Whitney test	***0.016***<br>***0.048***	*0.830*<br>*0.291*	*0.566*<br>*0.235*	*0.651*<br>*0.557*
**DIV 10-20**				
Control (4)	1791 ±128 (34)<br>34.6 ± 2.3	405 ± 32<br>8.3 ± 0.6	**1838 ± 129** (39)<br>**33.5 ± 2.7**	337 ± 25<br>7.2 ± 0.5
CNO	1672 ± 80 (54)<br>31.1 ± 1.7	409 ± 20<br>8.6 ± 0.4	**1306 ± 66** (41)<br>**25.9 ± 1.8**	349 ± 20<br>8.0 ± 0.5
Mann-Whitney test	*0.706*<br>*0.257*	*0.578*<br>*0.579*	***0.001***<br>***0.041***	*0.562*<br>*0.228*
**DIV 15-20**				
Control (2)	1836 ± 162 (22)<br>37.1 ± 2.5	330 ± 32<br>7.9 ± 0.7	**1684 ± 131** (17)<br>**34.8 ± 3.3**	314 ± 28<br>7.7 ± 0.7
CNO	1872 ± 114 (15)<br>31.3 ± 3.0	337 ± 43<br>7.4 ± 0.8	**1219 ± 131** (16)<br>**25.4 ± 2.0**	275 ± 21<br>6.3 ± 0.5
Mann-Whitney test	*0.653*<br>*0.170*	*0.956*<br>*0.485*	***0.004***<br>***0.049***	*0.276*<br>*0.189*
